# Photodynamic Therapy of Tumors Can Lead to Development of Systemic Antigen-Specific Immune Response

**DOI:** 10.1371/journal.pone.0015194

**Published:** 2010-12-10

**Authors:** Pawel Mroz, Angelika Szokalska, Mei X. Wu, Michael R. Hamblin

**Affiliations:** 1 Wellman Center for Photomedicine, Massachusetts General Hospital, Boston, Massachusetts, United States of America; 2 Department of Dermatology, Harvard Medical School, Boston, Massachusetts, United States of America; 3 Department of Immunology, Medical University of Warsaw, Warsaw, Poland; 4 Harvard-MIT Division of Health Sciences and Technology, Cambridge, Massachusetts, United States of America; University of Nebraska Medical Center, United States of America

## Abstract

**Background:**

The mechanism by which the immune system can effectively recognize and destroy tumors is dependent on recognition of tumor antigens. The molecular identity of a number of these antigens has recently been identified and several immunotherapies have explored them as targets. Photodynamic therapy (PDT) is an anti-cancer modality that uses a non-toxic photosensitizer and visible light to produce cytotoxic reactive oxygen species that destroy tumors. PDT has been shown to lead to local destruction of tumors as well as to induction of anti-tumor immune response.

**Methodology/Principal Findings:**

We used a pair of equally lethal BALB/c colon adenocarcinomas, CT26 wild-type (CT26WT) and CT26.CL25 that expressed a tumor antigen, β-galactosidase (β-gal), and we treated them with vascular PDT. All mice bearing antigen-positive, but not antigen-negative tumors were cured and resistant to rechallenge. T lymphocytes isolated from cured mice were able to specifically lyse antigen positive cells and recognize the epitope derived from beta-galactosidase antigen. PDT was capable of destroying distant, untreated, established, antigen-expressing tumors in 70% of the mice. The remaining 30% escaped destruction due to loss of expression of tumor antigen. The PDT anti-tumor effects were completely abrogated in the absence of the adaptive immune response.

**Conclusion:**

Understanding the role of antigen-expression in PDT immune response may allow application of PDT in metastatic as well as localized disease. To the best of our knowledge, this is the first time that PDT has been shown to lead to systemic, antigen- specific anti-tumor immunity.

## Introduction

To destroy tumors the immune system uses cytotoxic T-lymphocytes (CTLs) that recognize tumor antigens presented by major histocompatibility complex (MHC) class I molecules on the surface of tumor cells [1]. The molecular identity of a number of these antigens has been recently defined both in mouse and human tumors [2]. The tumor antigens identified to date have been broadly divided into following major groups [3]: (i) antigens encoded by cancer-testis genes expressed in various tumors, but not in normal tissues, such as the mouse gene *P1A* and human genes of the *MAGE, BAGE* and *GAGE* families [4], [5], [6], [7], [8], [9]; (ii) differentiation antigens of the melanocytic lineage, which are present on most melanomas but also on normal melanocytes [9], [10], [11]; and (iii) antigens that result from tumor-specific mutations in genes which are expressed in all tissues or come from viruses [12], [13], [14], [15], [16]. The immunotherapeutic strategies that target tumor antigens have been successfully developed and tested in preclinical studies and early-phase clinical trials [17], [18].

Photodynamic Therapy (PDT) uses a non-toxic dye molecule or photosensitizer (PS) that when activated by absorbed photon of light produces cytotoxic reactive oxygen species (ROS) [19]. Direct tumor killing by ROS, tumor-associated vascular damage and most notably activation of inflammatory responses make PDT an effective anti-cancer procedure [20], [21]. To date PDT has been approved by US Food and Drug Administration for use in bronchial and esophageal cancer and other premalignant and ophthalmological conditions [22]. Moreover, several other cancers are under active investigation [23].

PDT is thought to be particularly effective at stimulating an immune response against a locally treated tumor [20] for the following reasons. PDT has been shown to effectively engage both innate and adaptive immune systems in the host's responses to cancer [24], [25], [26]. PDT alters the tumor microenvironment by stimulating the release or expression of various pro-inflammatory and acute phase response mediators from the PDT-treated site [27], [28], [29], [30]. The body recognizes the presence of local trauma threatening the integrity of the affected site, and releases proinflammatory mediators to maintain homeostasis [31]. PDT thereby prompts a powerful acute inflammatory response, causing accumulation of neutrophils and other inflammatory cells in large numbers at the treated site and attack tumor cells [28], [32]. The activation of complement system has in particular emerged as a powerful mediator of PDT anti-tumor effects [33], [34], [35], [36], [37]. Complement not only acts as a direct mediator of inflammation but also stimulates cells to release secondary inflammatory mediators, including cytokines IL-1β, TNF-α, IL-6, IL-10, G-CSF, thromboxane, prostaglandins, leukotrienes, histamine, and coagulation factors [30].

In addition to stimulating local inflammation, PDT acts systemically to induce a potent acute phase response. PDT may also mature and activate dendritic cells and increase their ability to home to lymph nodes and efficiently present tumor antigens and prime lymphocytes [38].

The successful use of PDT to induce an effective local inflammatory response has been demonstrated in several tumor models [20], [39]; however, there is a limited amount of data recognizing the systemic immunological effects of this local treatment. In particular, the dependence and involvement of PDT mediated immunity on expression of tumor antigens has not been thoroughly established. We used a pair of equally lethal BALB/c colon adenocarcinomas, CT26 wild-type (CT26WT) and CT26.CL25 that expressed a tumor antigen, β-galactosidase (β-gal) to show that PDT treatment can elicit a systemic antigen/epitope specific anti-tumor immune response sufficiently robust to lead to regression of distant, well-established, antigen positive tumors outside the treatment field.

## Results

### PDT treatment leads to cures of antigen expressing tumors

**Figure 1:**
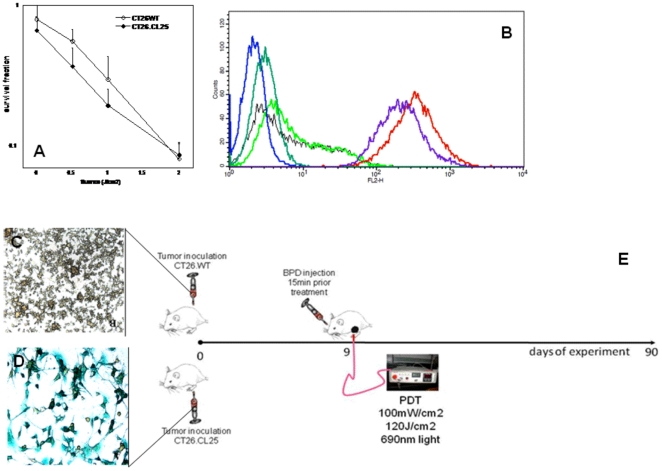
In vitro studies. A. *In vitro* PDT effectiveness against CT26WT and CT26.CL25 cells. The bars represent standard deviation. B. Histogram analysis of the levels of MHC I molecules in CT26.CL25 and CT26WT cell lines. (Blue) CT26.CL25 unstained control, (Dark Green) CT26.CL25 Isotype control, (Purple) CT26.CL25 anti-MCH I, (Black) CT26WT unstained control, (Bright Green) CT26WT Isotype control, (Red) CT26WT anti MHC I. C. Expression levels of β-gal antigen in CT26WT. D. Expression levels of β-gal antigen in CT26.CL25. E. Scheme of *in vivo* PDT.

The employed pair of previously described tumors, namely the β-gal antigen positive CT26.CL25 and antigen negative counterpart CT26WT cells were characterized by similar *in vitro* susceptibility to PDT (Figure 1A) and comparable levels of MHC class I molecules (Figure 1B). The CT26.CL25 cells displayed uniform expression of β-gal antigen (Figure 1C), while the CT26WT were β-gal antigen negative (Figure 1D).

**Figure 2:**
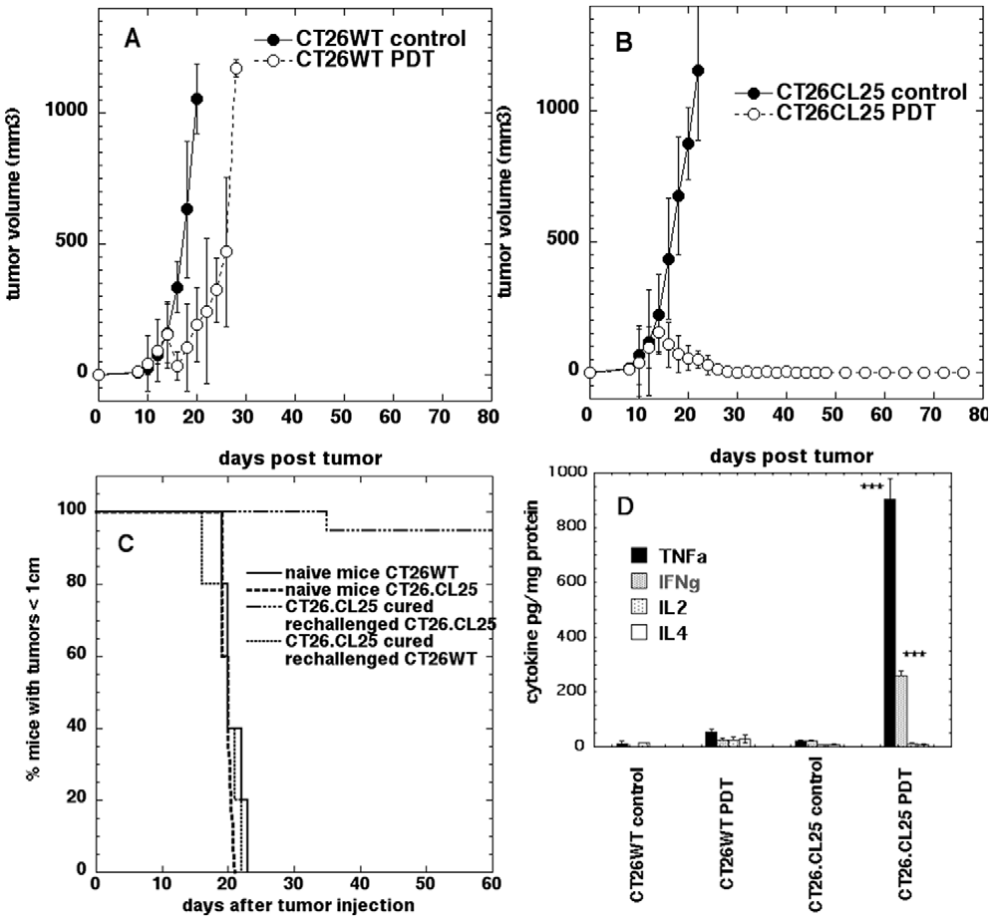
In vivo PDT of tumors (1 leg model). A. Plots of mean tumor volumes in mice bearing CT26WT tumors and B. CT26.CL25 tumors. Points are means of from 10–15 tumors and bars are SD. C. Kaplan-Meier survival curves of the % of mice cured from CT26.CL25 tumors and rechallenged either with CT26.CL25 cells or CT26WT cells. Naïve mice are included as a control for tumorigenic properties of the cells. Survival curve for rechallenge with CT26.CL25 cells is significantly different from the other two curves (P<0.0001). D. Mean levels of cytokines (TNF-alpha, IFN-gamma, IL-2 and IL-4) measured in the CT26WT and CT26.CL25 tumors 5 days after PDT as well as in control, non treated tumors. *** p<0.001. The bars represent standard deviation.

The scheme of the subsequent set of *in vivo* experiments is depicted in Figure 1E. We employed a vascular PDT regimen that was highly effective in mediating local regression in treated tumors. PDT produced a local response in all β-gal antigen negative CT26WT tumors as manifested by a marked reduction in size lasting until day 18 (Figure 2A). However, local tumor regrowth occurred relatively quickly and the net result was a growth delay of only 8–10 days. In marked contrast were the tumor volumes of β-gal antigen positive CT26.CL25 tumors treated with PDT (Figure 2B). The reduction in size was complete beyond day 20 and most importantly 100% of these PDT treated antigen positive tumors stayed in remission for the whole 90-day course of observation. Consequently, mice were declared cured according to the protocol.

To exclude the possibility that the observed difference in anti-tumor PDT effectiveness between CT26WT and CT25.CL25 could be attributed to the effects of the vector used to induce expression of β-gal, we created another control cell line CT26neo and Figure S1 shows the results of PDT treatment of this specially designed, additional, vector alone control.

### PDT cured mice reject rechallenge in an antigen specific manner

To assess memory immunity we performed rechallenge experiments. Mice bearing antigen positive CT26.CL25 tumors that had received PDT treatment and remained tumor free for 90 days were subsequently inoculated with the same antigen positive CT26.CL25 cells into the contralateral thigh. To assess the antigen specificity of the memory immunity some of the mice that were cured from CT26.CL25 cells were inoculated with antigen negative CT26WT cells. More than 95% of mice rechallenged with CT26.CL25 tumors rejected the tumor challenge and stayed tumor free for another 60 days of observation (Figure 2C), while all antigen negative CT26WT tumors progressed. Control survival curves of naïve mice bearing CT26.CL25 or CT26WT tumors demonstrated that the cells used for rechallenge retained their full tumorigenic potential.

### PDT treatment leads to increase in local production of TNFα and IFNγ cytokines in antigen positive tumors

We assessed the extent of local activation of the immune system by measuring secreted cytokines in the tumor. We observed that PDT treatment of antigen positive CT26.CL25 (but not antigen-negative CT26WT) tumors led to striking and significant (p<0.001) increases in tumor necrosis factor alpha (TNF**α**) and interferon gamma (IFN**γ**) levels (Figure 2D) suggesting the active involvement of the Th1 arm of adaptive immune response. The production of IL-2 and IL-4 was not significantly different from the non-treated control levels.

### PDT induced cytotoxic T cells specifically destroy antigen-positive cancer cells

**Figure 3:**
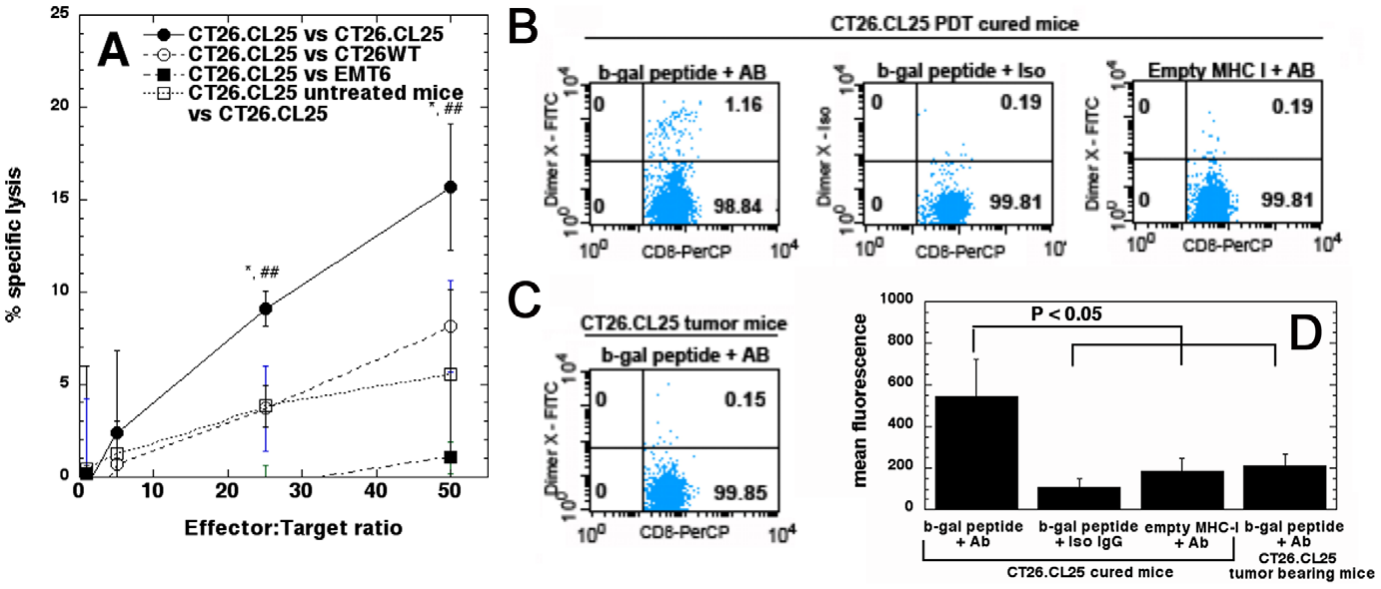
Analysis of antigen and epitope specificity of observed PDT induced immune response. A. Percentage of specific lysis of target cells (CT26.CL25, CT26WT or EMT6 as an irrelevant target control) by CTLs isolated from either CT26.CL25 PDT cured or control CT26.CL25 tumor bearing mice (3–4 mice per group). Data are representative of 3 independent experiments. * P<0.05 compared to CT26.CL25 cured CTLs against CT26WT targets, and compared to CTLs from CT26.CL25 tumor bearing mice. ## P<0.001 compared to CT26.CL25 cured CTLs against EMT6 targets. The bars represent standard deviation. B. Lymph node cells isolated from PDT treated mice curedfrom antigen positive CT26.CL25 tumors 5 days after rechallenge incubated with DimerX loaded with TPHPARIGL peptide derived from β-gal antigen or empty DimerX, and either FITC-detection antibody or FITC isotype control. Additionally cells were stained for CD8 expression to assess percentage of CD8-DimerX-FITC double positive cells. C. Lymph node cells from CT26.CL25 control tumor bearing mice incubated with DimerX loaded with TPHPARIGL peptide derived from β-gal antigen and FITC-detection antibody. Additionally cells were stained for CD8 expression to assess percentage of CD8-DimerX-FITC double positive cells. D. Quantification and statistical analysis of the FACS plots described above (6 mice per group). The bars represent standard deviation.

To confirm that PDT leads to development of β-gal antigen specific cytotoxic T cells able to specifically lyse tumor cells in an antigen specific manner we used ^51^Cr release assay. We harvested the regional, tumor draining lymph nodes from CT26.CL25 cured mice five days after tumor rechallenge as well as from control, tumor-bearing mice 9 days after tumor inoculation. Figure 3A shows that CTLs from mice cured from antigen positive CT26.CL25 tumors with PDT displayed significantly more specific lysis at effector to target ratios of 25∶1 and 50∶1 against CT26.CL25 targets than they did against antigen negative CT26WT targets (P<0.05) or irrelevant, antigen negative EMT6 targets (P<0.001). Likewise, lymphocytes from CT26.CL25 tumor bearing mice also showed significantly less specific lysis against CT26.CL25 targets than did CTLs from CT26.CL25 PDT cured mice (P<0.05).

### PDT elicits development of epitope specific CD8+ T cells

In order to demonstrate that PDT of antigen positive CT26.CL25 tumors can lead to recognition of specific epitopes derived from particular tumor antigen we used Dimer X staining [40]. The lymph node cells isolated from mice either cured from antigen positive CT26.CL25 mice (Figure 3B) or control, non-treated CT26.CL25 tumor bearing mice (Figure 3C) were incubated with DimerX loaded with nonapeptide derived from β-gal antigen (TPHPARIGL peptide). There was a significant difference (Figure 3D) between binding of β-gal loaded DimerX by CD8 positive T cells isolated from mice cured from CT26.CL25 tumors and from naïve CT26.CL25 tumor bearing mice. These results show that PDT does indeed induce recognition of MHC class I bound epitope derived from β-gal antigen and provide an explanation for the specific cell lysis found in the chromium release experiments.

### PDT of antigen-positive tumors leads to destruction of distant, untreated, established, antigen-positive tumors

**Figure 4:**
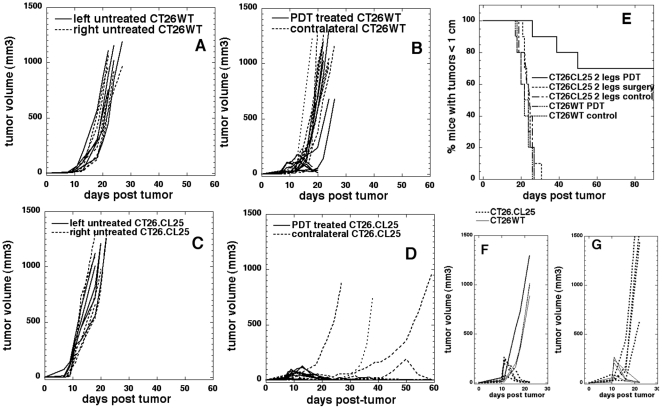
In vivo PDT of tumors (2 leg model). Time courses of individual tumor volumes in mice with two similar bilateral or mismatched tumors in right and left legs. A. Bilateral CT26WT tumors, right leg treated with PDT (n = 10); B. Bilateral CT26WT tumors, untreated (n = 5); C. Bilateral CT26.CL25 tumors, right leg treated with PDT (n = 10); D. Bilateral CT26.CL25 tumors, untreated (n = 5). E. Kaplan-Meier survival curves of the % of mice with tumors less than 1-cm diameter in five groups of mice. Three groups had two similar bilateral CT26.CL25 tumors (one group was untreated, one group had right leg tumor treated with PDT and one group had right leg tumor surgically removed). Two further groups had two bilateral CT26WT tumors (one group was untreated, and the other group had the right leg tumor treated with PDT). The survival curve of the mice with bilateral CT26.CL25 tumors treated with PDT is significantly different from the other survival curves (P<0.0001). F. Mismatched tumors. CT26WT and CT26.CL25 tumors, CT26WT treated with PDT (n = 5). G. Mismatched tumors. CT26WT and CT26.CL25 tumors, CT26.CL25 treated with PDT (n = 5).

To further evaluate whether PDT treatment can elicit β-gal antigen specific systemic immune response strong enough to destroy distant, established, non-treated tumors we performed PDT in mice bearing two bilateral tumors. In this model only one tumor was illuminated, while the contralateral tumor was shielded from light. The antigen negative CT26WT tumors that received treatment could not be followed for the long-term outcome because all untreated, contralateral tumors continued their growth uninterrupted, confirming the lack of any PDT induced anti-tumor immunity (Figure 4B). The untreated control bilateral CT26WT tumors grew equally well leading to mouse sacrifice when tumors reached 1 cm in diameter (Figure 4A). Mice bearing β-gal antigen positive CT26.CL25 tumors in both legs, which had only one tumor illuminated, demonstrated a remarkable and interesting response: the PDT treated tumors regressed in all cases; in 9 out of 10 mice the distant untreated tumors also shrank and disappeared for at least 20 days, while in one mouse the tumor continued growth unabated. In 6 out of 9 mice the tumor regression of their contralateral tumors lasted beyond day 20 and was permanent. In 2 out of 9 mice the untreated, contralateral tumors recurred about day 30 and in one mouse the untreated, contralateral tumor regrew briefly about day 50 before also regressing permanently. The growth curves of these tumors are shown in Figure 4D and the corresponding growth curves of untreated bilateral CT26.CL25 tumors are shown for control purposes in Figure 4C. Kaplan-Meier curves for the percentage of mice with both tumors smaller than 1-cm in diameter are shown in Figure 4E.

It was considered possible that the simple mechanical removal of one of the tumors by the ablative function of PDT could affect the growth of the contralateral one. To test and exclude this possibility a group of mice bearing bilateral antigen positive CT26.CL25 tumors had the right-leg tumor surgically removed at the same time as PDT was carried out to other groups, but this treatment had no effect on the progression of the contralateral tumors (Figure 4E). The survival curve for the CT26.CL25 PDT treated group was significantly different from all other experimental groups (P<0.0001, log-rank test).

To further investigate whether observed destruction of contralateral, established, non-treated tumors was antigen specific we carried out experiments with two groups of mice that each had two mismatched tumors, antigen negative CT26WT in left leg and antigen positive CT26.CL25 in right leg. One group had only CT26WT tumors treated with PDT and the other group had only CT26.CL25 tumors treated with PDT. The PDT treated tumors showed the expected PDT response (more pronounced in the case of CT26.CL25), but since there were no effects on the size or growth rate of the contralateral untreated tumors in either case (Figure 4 F&G) mice could not be followed for long-term outcome.

### Activated cytotoxic T cells infiltrate antigen-positive PDT treated and non-treated, contralateral tumors

**Figure 5:**
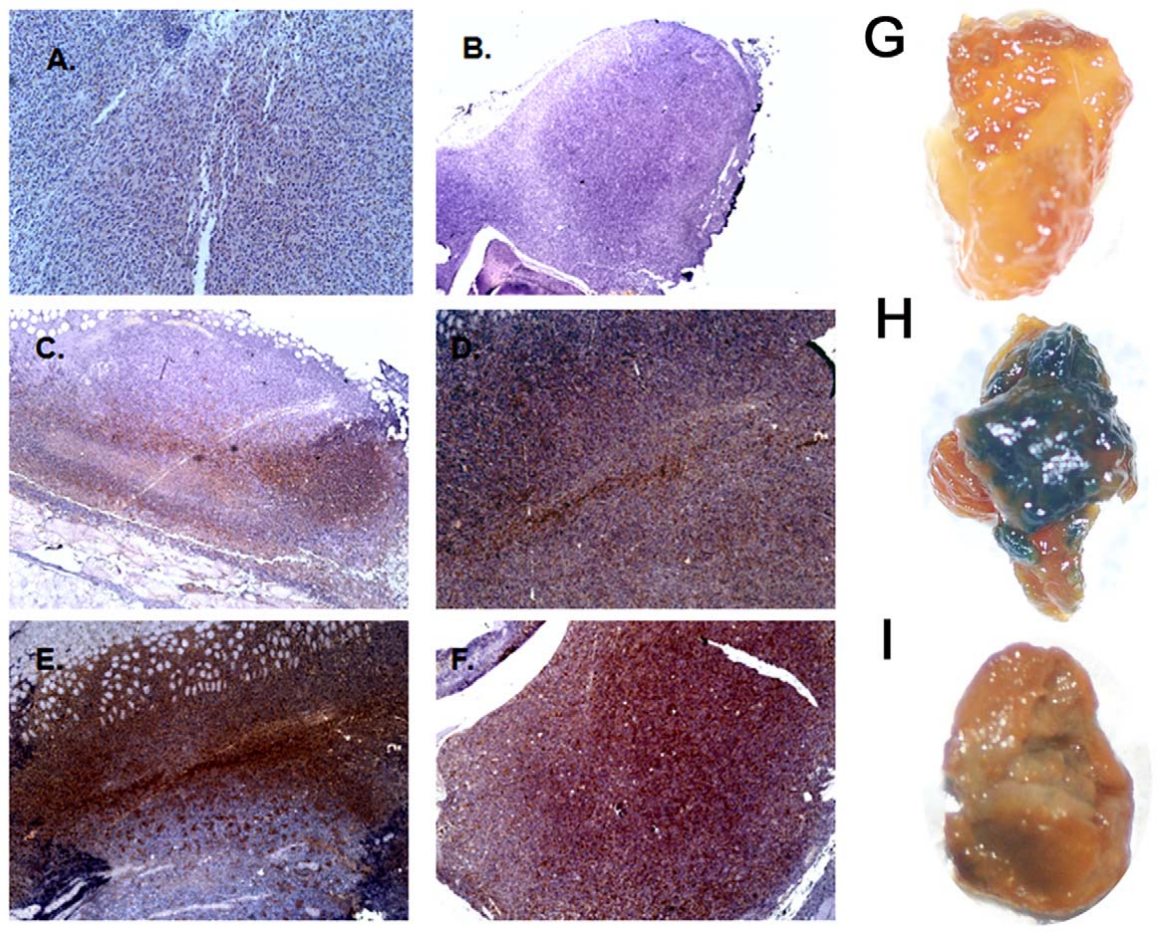
Immunohistochemical staining for LAMP-1 (CD107a) of CT26.CL25 tumors. A. non-treated control. B. negative control for staining. C. CT26.CL25 PDT treated tumors day 5. D. CT26.CL25 non-treated, contralateral tumors day 5. E. CT26.CL25 PDT treated tumors day 16. F. CT26.CL25 non-treated, contralateral tumors day 16. Analysis of β-gal antigen expression and loss by X-gal staining. G. CT26WT control tumors negative for β-gal antigen, H. CT26.CL25 non-treated control tumors which show robust blue positive staining for β-gal antigen. I. CT26.CL25 non-treated, contralateral tumors that escaped immune surveillance and continued to grow. They show significantly decreased staining for β-gal antigen.

To further confirm the involvement of the immune system in the observed PDT response and the destruction of contralateral, established, non-treated tumors we performed immunohistochemical staining for LAMP-1 (CD107a) presence as a marker for intratumoral activated cytotoxic T cell infiltration [41], [42]. We observed that CT26.CL25 non-treated, antigen positive control tumors demonstrated some staining (Figure 5A), while PDT treated CT26.CL25 tumors examined 5 and 16 days after PDT treatment revealed pronounced T cell infiltration (Figure 5C&E). In addition, the LAPM-1 staining demonstrated that contralateral (Figure 5D&F) antigen positive CT26.CL25 tumors are also heavily infiltrated by LAMP-1 positive T cells. Moreover, there was noticeable increase in T cell infiltration/LAMP-1 staining between day 5 and 16 which corresponded well with the observed decrease in tumor size.

### Tumors escape PDT mediated immune surveillance by decreasing antigen expression

It was possible that the reason why 3 out of 10 contralateral tumors escaped from PDT mediated immune recognition and elimination could be due to the loss of the expression of the β-gal antigen under the pressure of immune destruction. We therefore harvested the contralateral tumors that progressed after PDT and stained them for β-gal antigen presence. We observed that indeed tumors which escaped immune destruction had significantly lower levels of β-gal antigen (compare Figures 5H&I).

### Lack of adaptive immune system abrogates PDT anti-tumor effects

**Figure 6:**
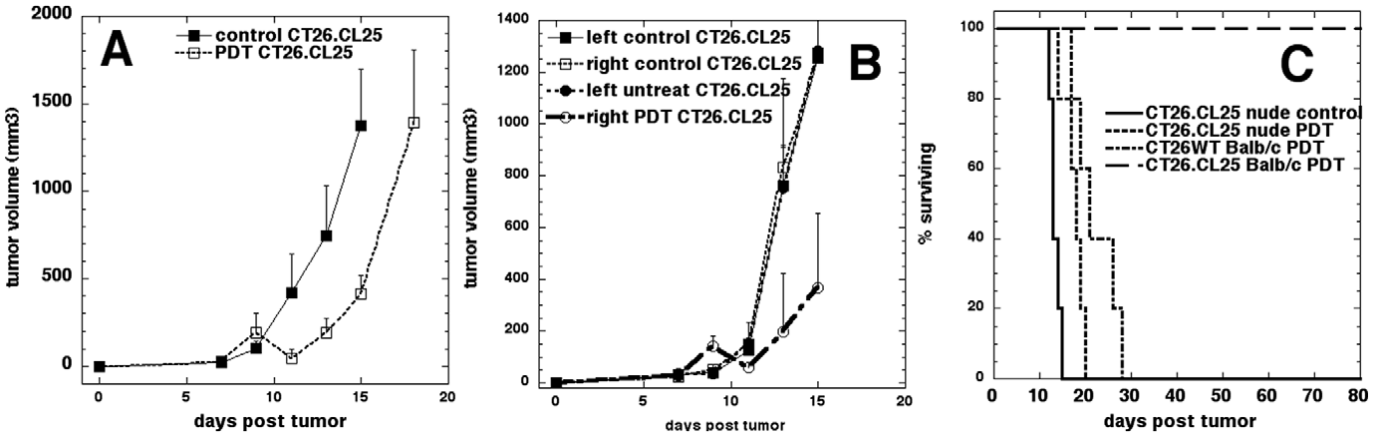
Lack of adaptive immune response abrogates PDT anti-tumor effects. A. Tumor volumes of CT26.CL25 tumors subjected or not to PDT in BALB/c Nu/Nu immunocompromised mice. The bars represent standard deviation. B. Tumor volumes of bilateral CT26.CL25 tumors subjected or not to PDT in BALB/c Nu/Nu immunocompromised mice. The bars represent standard deviation. C. Kaplan-Meier analysis comparing the % of surviving BALB/c and BALB/c Nu/Nu mice bearing CT26.CL25 of CT26WT tumors subjected to PDT. Non-treated BALB/c Nu/Nu mice bearing CT26.CL25 are included for control (n = 5).

To confirm that the observed PDT effects are indeed due to the activation of the immune system we repeated the experiments with β-gal antigen positive CT26.CL25 tumors in immunocompromised BALB/c Nu/Nu mice. In a one-leg model PDT produced a local response similar to that observed in antigen negative CT26WT tumors, but no permanent cures were observed (Figure 6A). To provide additional evidence for the involvement of the immune system in the destruction of the non-treated, contralateral, β-gal antigen positive CT26.CL25 tumors, we also repeated the PDT experiments in immunocompromised mice bearing bilateral CT26.CL25 tumors. As can be seen in Figure 6B, PDT treatment provided good local response, but it did not affect the growth of the non-treated, contralateral, β-gal antigen positive CT26.CL25 tumors. In figure 6C we compared survival of immunocompetent BALB/c and immunocompromised BALB/c Nu/Nu mice bearing CT25.CL25 as well as CT26WT tumors. As can be seen PDT treatment of CT25.CL25 tumors in immunocompetent mice resulted in 100% survival. However, the PDT treatment of CT26.CL25 tumors growing in immunocompromised mice failed to produce any cures and the recurrence of CT25.CL25 tumors in BALB/c Nu/Nu mice closely resembled the recurrence of CT26WT tumors in BALB/c mice. These results provide strong evidence that the curative effects observed in case of antigen positive CT26.CL25 tumors were mediated by PDT activated, antigen specific immune response, and that the lack of functional adaptive immune system abrogates this effect.

## Discussion

In this study we have employed a pair of previously described tumors, CT26.CL25 transduced with lacZ gene to stably express a model tumor antigen (β-gal) and its wild type counterpart CT26. This pair of tumors allowed us to design a study model closely resembling the clinical situation to investigate the importance of the antigen presence and differences of PDT-induced immune reaction between antigen expressing and antigen-negative cancer cell lines, otherwise being identical. In this model both wild type and β-gal tumors were equally lethal, suggesting that the level of β-gal expression was low enough to allow tumor to grow without triggering any clinically significant immune response, a situation often observed in cancer patients [43]. It was only when PDT was applied that the significant differences in the therapeutic outcome and the elicited immune response were observed.

The present study shows that PDT can induce a highly potent antigen specific immune response capable of inducing memory immunity that enables mice to reject a tumor rechallenge with the same antigen positive tumor from which they were cured. The *in vivo* PDT-induced immune response led to an increased release of TNFα and IFNγ cytokines within the treated tumors. The CTLs from PDT treated mice bearing antigen positive CT26.CL25 tumors were capable of causing specific lysis of antigen positive target cells and bind the immunodominant peptide epitope derived from β-gal antigen restricted by MHC class I haplotype H2L^d^. PDT induced immune response was also capable of causing regression of distant established tumors that received no treatment. The robust infiltration of PDT treated and non-treated, contralateral tumors by activated, antigen specific effector CTLs has also been confirmed. However, the regression of distant tumors occurred in only 70% of mice and the reason why some tumors escaped from immune recognition and elimination was shown to be the loss of expression of the tumor antigen. This is the first time that the escape from PDT induced immune surveillance due to the loss of tumor antigen has been demonstrated. However, this phenomenon has been previously described [44], [45], [46], [47] also in the case of CT26.CL25 model [48] when lung metastases from CT26.CL25 tumors, which had escaped from immune control after virus-mediated vaccination, were shown to have reduced β-gal activity. Most importantly we showed that the observed PDT anti-tumor effects are completely abolished when there is no functional adaptive immune response.

Our findings are in accordance with recently published study by Kabingu *et al.* [49] where it was shown that PDT could lead to immune recognition of hedgehog-interacting protein 1 (Hip1) antigen in patients with basal cell carcinoma. These observations show that PDT has the capability to be a local cancer therapy that could be usefully applied even when the primary tumor has spread at the time of treatment. The fact that this potent immune response was only observed in the antigen positive tumors emphasizes the importance of the presence of a tumor antigen capable of being efficiently recognized by cytotoxic effector T cells. These data demonstrate that PDT induced CTLs discriminate and target antigen expressing tumor as well as that PDT immunity depends on the presence of tumor antigen.

The expression of β-gal antigen in mouse tumors has been previously described and it was found that in certain circumstances it could act as a potent immunostimulant leading to generation of cytotoxic T lymphocytes [50], [51]. The reported antitumor effects of vaccination protocols with vectors carrying the β-gal gene had to be significantly enhanced by the co-administration of certain cytokines [50] or by the viral vectors simultaneously encoding cytokine genes [52]. The active immunity against established tumor produced by vaccinationin the absence of additional interventions, failed to have an impact on tumor burden. Therapeutic responses in tumor bearing animals could only be improved however, when particular cytokines (rhIL-2, rmIL-6, rhIL-7, and rmIL-12) were given following vaccine administration. However, it has never before been reported that a single immunotherapy treatment of the β-gal positive tumor can lead to total tumor rejection [53]. Our data strongly suggest that applying a single PDT treatment to an antigen-expressing tumor may not only destroy the primary tumor, but also induce a systemic immune response capable of destroying distant antigen positive metastases.

One interesting observation from our study that needs explanation is the failure of PDT to induce anti-tumor immunity in CT26WT tumors. PDT of CT26 tumors growing in immunocompetent mice followed by intratumoral injection of immature dendritic cells has been previously shown to produce some immune response leading to a slower growth of a tumor rechallenge, and this combination therapy was able to generate CTLs capable of lysing tumor cells *ex vivo* [21], [38], [54]. There are also several reports that CT26 tumors in its wild-type state do express tumor antigens [55] in particular a single peptide known as AH-1, a non-mutated nonamer derived from the envelope protein (gp70) of an endogenous ecotropic murine leukemia provirus [55]. Nevertheless, most authors agree that in practice CT26WT tumors in BALB/c mice generally evade the immune response due to the presence of CD4+CD25+ T-regulatory suppressor cells [56], down-regulation of gp70 production [57], or the presence of immune tolerance [58]. Recently, a paper by McWilliams *et al*. [59] described the expression of *gp70* mRNA in several tissues of BALB/c mice resulting in immunologic tolerance that affects antitumor immunity. In view of these reports it is possible that gp70 antigen in CT26WT tumors behaves like self antigen and therefore an intervention targeting T regulatory cells may potentiate PDT immune response in this model. We have previously shown that low dose cyclophosphamide (CY) can deplete T regulatory cells and augment PDT immunity in J774 tumors [60] and we subsequently found out that the combination of the described low-dose regimen of CY and PDT may lead to significant numbers of cures in the CT26WT model (unpublished data).

The employed tumor antigen model somewhat differs from many naturally occurring cancer antigens, including the fact that the expression of the antigen is limited to tumor tissue or that the immune response is studied in established transplanted tumors and not in metastatic setting. Many would argue that the true test of a systemic immune response is the effects on distant metastases. However, there are reports [61], [62] where the effectiveness of immunotherapy was demonstrated both in models of metastatic disease and in animals bearing established tumors. Furthermore, the destruction of an untreated established tumor may appear as a more severe test of active immune response than metastatic disease which, microscopic in nature, may be more easily penetrated by tumor specific immune cells. Notwithstanding with the restrictions of the selected model the presented results however, are a straightforward demonstration of the importance of tumor antigens in promoting immune rejection of tumors and in this regard they may have significant implications for the design of clinical protocols using PDT to treat human cancers. We believe that more investigators should consider whether antigen-specific immune response is involved in patients receiving PDT for cancer. Consequently, the results presented in this study have led us to explore the effects of PDT employing tumors expressing clinically relevant tumor antigens like P815 mastocytoma expressing murine homologue of cancer testis antigen *P1A* [63] or pancreatic adenocarcinoma Panc02 expressing human carcinoembryonic antigen [64]. The preliminary results obtained in these models are highly encouraging and in agreement with the presented data (unpublished results).

In conclusion, we have shown that an effective vascular PDT regimen that can reliably produce local tumor destruction can also induce potent, systemic, antigen specific anti-tumor immunity. The observed immunity was capable of causing regressions and cures in distant, established, antigen positive tumors outside the illumination field, and also of inducing long-term immune memory and resistance to rechallenge. This tumor-destructive effect was mediated by tumor antigen specific cytotoxic T-cells that recognize the immunodominant epitope of β-gal antigen and are induced after PDT. These data encourage clinical trials of PDT in patients with tumor types known to express tumor associated antigen (melanoma, renal cell carcinoma etc).

## Materials and Methods

### Cell lines

CT26 wild type (CT26WT) and CT26.CL25 cell lines (ATCC, Mannassas, VA). were cultured in RPMI medium with L-glutamine and NaHCO_3_ supplemented with 10% heat inactivated fetal bovine serum, penicillin (100 U/mL) and streptomycin (100 µg/mL) (all from Sigma, St Louis, MO) at 37°C in 5% CO_2_ in 75 cm^2^ flasks (Falcon, Invitrogen, Carlsbad, CA). CT26neo cell line was provided by Dr. Andrew Kung from Department of Pediatric Oncology, Dana-Farber Cancer Institute, Boston, MA. VSVG-pseudotyped retrovirus was packaged by triple transfection of pLNCX-neo, pMD-MLV, and pMD-G (Richard Mulligan, HHMI, Boston, MA) into 293T cells. CT26WT cells were infected with filtered retroviral stocks at a multiplicity of infection of 10 in the presence of 8 µg/ml of polybrene. CT26neo and CT26.CL25 cells were cultured in constant presence of 500 µg/mL G418 antibiotic (Sigma, St Louis, MO) in order to maintain constant expression of the vector.

### Cell X-gal staining

To detect β-gal antigen CT26.CL25, CT26WT and CT26neo cells were fixed with X-gal Fix buffer (0.1 M phosphate buffer (pH 7.3) supplemented with 5 mM EGTA (Sigma), pH 7.3, 2 mM MgCl_2_ and 0.2%glutareldahyde (Sigma)) for 15 min, than washed twice (5 minutes each) with X-gal Wash buffer (0.1 M phosphate buffer (pH 7.3) supplemented with 2 mM MgCl_2_). Next the X-gal staining buffer containing 1 mg/mL X-gal (0.1 M phosphate buffer (pH 7.3) supplemented with 2 mM MgCl2, 5 mM potassium ferrocyanide and 5 mM potassium ferricyanide was added and cells were incubated overnight.

### Tumor X-gal staining

We used a modified method of staining mouse tumors with X-gal [65]. Briefly to detect beta-gal expression non-treated CT26WT, CT26.CL25 and contralateral CT26.CL25 tumor samples that escaped PDT induced immunity were harvested when tumors reached 1.5 cm in diameter, fixed with Fix buffer (0.1 M phosphate buffer (pH 7.3) supplemented with 5 mM EGTA (Sigma), pH 7.3, 2 mM MgCl_2_ and 0.2% glutaraldahyde (Sigma)) for 15 min and washed twice (5 minutes each) with X-gal Wash buffer (0.1 M phosphate buffer (pH 7.3) supplemented with 2 mM MgCl_2_). Next the X-gal staining buffer containing 1 mg/ml of X-gal (0.1 M phosphate buffer (pH 7.3) supplemented with 2 mM MgCl_2_, 5 mM potassium ferrocyanide (K_4_Fe(CN)_6_-3H_2_O) and 5 mM potassium ferricyanide (K_3_Fe(CN)_6_) was added and tumors were incubated overnight at 37°C. The pictures were taken with the Olympus SLR digital camera.

### Flow cytometry analysis of MHC class I molecules levels

CT26.CL25 and CT26WT cells were fixed and incubated at room temperature for 1 h with PE-Conjugated anti H2-D^d^ antibody (BD Pharmingen). PE Isotype antibody and unstained cells were used as controls. Next cells were washed twice in 1 ml of PBS and analyzed on FACScalibur (BD).

### Photosensitizer and light source

Liposomal benzoporphyrin derivative mono acid ring A (Verteporfin for injection, BPD, QLT Inc, Vancouver, BC, Canada) and was prepared by diluting the powder to a concentration of 0.3 mg/mL in sterile 5% dextrose. A 1W 690-nm diode laser (B&W Tek Inc., Newark, DE) was coupled into a 0.8-mm diameter fiber and a lens was used to obtain a uniform spot.

### In vitro PDT

10000 CT26WT, CT26.CL25 and CT26neo cells were plated per well in 96 well plates and incubated for 1-h with 200 nM BPD. %. After incubation the medium was replaced with 200 µL of fresh medium and PDT was performed. 690 nm laser light dose was varied and fluences of 0 (dark toxicity) to 2 J/cm^2^ were delivered at an irradiance of 50 mW/cm^2^ to each well separately (4 wells represented a group). Controls entailed cells with no treatment and cells with light alone at the highest fluence or with photosensitizer alone. At the completion of the illumination, the plates were returned to the incubator for 24 h before initiating further studies. A 4-h MTT colorimetric assay [3-(4,5-dimethylthiazol-2-yl)-2,5-diphenyltetrazolium bromide] was used that measures mitochondrial reductase activity. This assay correlates well with colony-forming assays as a measure of cell viability, as has been described previously. The absorbance for MTT assay was read at 560 nm.

### Animal tumor model

BALB/c and BALB/c Nu/Nu mice (6–8 weeks old) were purchased from Charles River Laboratories (Boston MA). All experiments were carried out according to a protocol approved by the Subcommittee on Research Animal Care (IACUC) at MGH and were in accord with NIH guidelines. Mice were inoculated with 350,000 cells subcutaneously into the depilated right thigh. Two orthogonal dimensions (a and b) of the tumor were measured 2–3 times a week with vernier calipers. Tumor volumes were calculated as follows, volume  = 4π/3× [(a+b)/4]^3^. When tumors reached a diameter of 5–7 mm (9 days after inoculation) PDT was performed.

### PDT and tumor response

Tumor bearing mice were anaesthetized with i.p. injection of 87.5 mg/kg of ketamine and 12.5 mg/kg xylazine and BPD (1 mg/kg in 5% dextrose solution) was administered i.v. via the supraocular plexus. Control mice received 5% dextrose only. Fifteen minutes after BPD injection illumination was performed. A total fluence of 120 J/cm^2^ was delivered at a fluence rate of 100 mW/cm^2^. The mice were sacrificed when any of the tumor diameters exceeded 1.5 cm (1 cm diameter for 2 legs. and rechallenge models).

### Rechallenge

Mice surviving ninety days after PDT were subsequently rechallenged with 350,000 cells of CT26.CL25 or CT26WT in the contralateral thigh and monitored for another 60 days. Naïve control mice were inoculated with the same sample of cells to confirm tumorigenicity.

### Cytokine production in excised tumors

The levels of cytokines (TNF-alpha, IFN-gamma, IL-2 and IL-4) were measured in the CT26WT and CT26.CL25 tumors harvested 5 days after PDT as well as in control, non-treated tumors. We used a mouse cytokine capture bead assay (BDTM Mouse Th1/Th2 Cytokine Kit, Becton-Dickinson, San Diego, CA). The assays were performed according to the manufacturer's instructions. In brief, tumor samples were homogenized in glass homogenizer and centrifuged to collect supernatants. A 50 µL aliquot of the supernatant of each sample was stained with a suspension of mouse cytokine beads and the phycoerythrin (PE) detection reagent. After 2 hr of incubation, samples were washed and then analyzed by flow cytometry and CBA software (BD Biosciences). Mouse Th1/Th2 standards provided with the kit were appropriately diluted and used in parallel to samples for preparation of the standard curves. Two different samples were used for different tumors and each group was repeated twice.

### Lymphocyte preparation

Inguinal lymph nodes from tumor-immune mice sacrificed five days after CT26.CL25 rechallenge and from tumor-bearing mice sacrificed 9 days after tumor injection were homogenized with a pellet pestle (Kontes Glass Co, Vineland, NJ) and passed through a 70 µm mesh nylon cell strainer (BD Falcon) to make single cell suspensions.

### Chromium release assay

Cytotoxicity was measured by ^51^Cr release assay. Lymphocyte suspensions (0.1 mL) were dispensed to wells of U-bottom 96-well microtiter plates (six wells replicates for each effector:target (E∶T) ratio). One million target cells (CT26WT, CT26.CL25 or EMT6) were labeled for 2 h with 100-µCi of ^51^Cr (NEN Perkin Elmer, Waltham, MA), washed and then 10,000 target cells were mixed with effector CTLs at various E∶T ratios and incubated for 4-h at 37°C, 5% CO_2_. 40 µL of supernatant were mixed with 150 µL of scintillation cocktail (Optiphase Supermix. NEN Perkin Elmer) and a beta scintillation was read with plate reader (MicroBeta Trilux Model 1450, Wallac-Perkin-Elmer). The final percentage of specific lysis was calculated as follows: {test ^51^Cr released – spontaneous ^51^Cr released}/{maximum ^51^Cr released – spontaneous ^51^Cr released}. The maximal release was obtained by incubation of target cells in 0.5% SDS.

### Dimer X staining

The H2L^d^ specific β-gal peptide TPHPARIGL [51] was synthesized by the MGH Institutional Peptide Synthesis Core. 4 µg of soluble dimeric mouse H-2L^d^:Ig fusion protein (BD Biosciences, San Jose, CA) was loaded with a 640-fold molar excess of TPHPARIGL peptide overnight at 37°C. Lymphocytes from CT26.CL25 immune or tumor-bearing mice were incubated with staining cocktail (H-2L^d^:Ig peptide loaded, PerCP labeled anti-CD8a clone 53-6.7 (BD Pharmingen) and FITC-labeled rat anti-mouse IgG1 clone A85-1 (BD Biosciences) for 1 h at room temp, washed and FACS analysis was performed. (FACScalibur, BD Biosciences). The appropriate FITC and PerCP secondary antibody isotype controls as well as staining with empty H-2L^d^:Ig fusion protein was performed. FACS scattergrams were analyzed by first gating for size and CD8 expression on FL3 vs FSC dot plot and next by re-plotting CD8 positive cells on FL1 vs FL3 dot plot to assess percentage of CD8-DimerX-FITC double positive cells.

### PDT in bilateral tumor model

Mice were inoculated in both legs either with CT26WT, CT26.CL25 or one CT26WT and one CT26.CL25 tumors and two equal sized tumors reliably grew. After BPD injection only one tumor received light treatment. Ten mice with bilateral CT26.CL25 tumors had their right legs amputated above the tumor at the same day as PDT was performed. This procedure was done under anesthesia, using a scalpel, the femoral artery was isolated, sutured and cut with VICRIL 7-0 synthetic absorbable suture (ETHICON INC. NJ), the bone was cut after hemostasis. The wound was closed in two planes, first muscle using VICRIL 7-0 suture, the second plane was skin closed using black monofilament Nylon nonabsorbable surgical suture (ETHICON, INC). All these procedures were done following aseptic techniques.

### Immunohistochemistry

Formalin-fixed, paraffin-embedded sections of non-treated control, PDT treated and non-treated contralateral CT26.CL25 tumors harvested on day 5 and 16 of the experiment were sectioned serially (5 µm). Slides were deparaffinized, subjected to heat-based antigen retrieval (BD Pharmingen) and stained with Vectorstain ABC kit (Vector Laboratories, Inc, Burlingame, CA). First slides were incubated with 10% normal rabbit serum for 20 minutes at room temperature (RT) and next a 1∶200 dilution of rat monoclonal anti LAMP-1 antibody in 1% BSA in PBS (Santa Cruz; Santa Cruz, CA) was added for overnight incubation at 4°C. For negative staining control 1% BSA in PBS was used instead of primary antibody. On the following day a biotinylated secondary rabbit anti-rat antibody was added for 30 minutes at RT and following wash with PBS ABC reagent was added for 45 minutes at RT. Staining was visualized using Vector *Nova*Red, and hematoxylin was used as a nuclear counterstain.

### Statistics

All values are expressed as ± standard deviation and all experiments were repeated at least twice with comparable results. Differences between means were tested for significance by one-way ANOVA. Survival analysis was performed using the Kaplan-Meier method. P-values of <0.05 were considered significant.

## Supporting Information

Figure S1 **Tumor volumes of CT26neo tumors subjected or not to PDT.** Due to variable response of CT26neo tumors to PDT tumor volumes of individual mice in the PDT group are presented. The one mouse that was cured from CT26neo failed to reject a rechallenge with CT26neo (data not shown).
(DOC)
