# Supplementary material for: Photodynamic Therapy of Tumors Can Lead to Development of Systemic Antigen-Specific Immune Response
Source: PLoS One. 2010 Dec 10;5(12):e15194. doi: 10.1371/journal.pone.0015194 (PMC3001867; doi:10.1371/journal.pone.0015194)
Supplement: Figure S1 — Tumor volumes of CT26neo tumors subjected or not to PDT. Due to variable response of CT26neo tumors to PDT tumor volumes of individual mice in the PDT group are presented. The one mouse that was cured from CT26neo failed to reject a rechallenge with CT26neo (data not shown). (DOC) [file pone.0015194.s001.doc]

**Supplementary Materials.**

**Figure S1.** Tumor volumes of CT26neo tumors subjected or not to PDT. Due to variable response of CT26neo tumors to PDT tumor volumes of individual mice in the PDT group are presented. The one mouse that was cured from CT26neo failed to reject a rechallenge with CT26neo (data not shown).
